# Developing and Implementing the Family Nurse Practitioner Role in Eswatini: Implications for Education, Practice, and Policy

**DOI:** 10.5334/aogh.2813

**Published:** 2020-05-18

**Authors:** Colile P. Dlamini, Thembisile Khumalo, Nkosinathi Nkwanyana, Tengetile R. Mathunjwa-Dlamini, Liz Macera, Bonisile S. Nsibandze, Louise Kaplan, Eileen M. Stuart-Shor

**Affiliations:** 1 Faculty of Health Sciences, University of Eswatini, SZ; 2 Kingdom of Eswatini Ministry of Health, SZ; 3 Eswatini Nursing Council, SZ; 4 Washington State University College of Nursing Vancouver, US; 5 Tumwater Family Practice Clinic, Tumwater, WA, US; 6 University of Massachusetts Boston, College of Nursing and Health Sciences, US; 7 Department of Anaesthesia, Critical Care and Pain Medicine, Beth Israel Deaconess Medical Centre, US

## Abstract

**Introduction:**

Eswatini, a small, largely rural country in Southern Africa, has a high burden of morbidity/mortality in the setting of a critical shortage of human resources for health. To help achieve universal access to healthcare across the lifespan, the advanced practice family nurse practitioner (FNP) role was proposed and is in the process of being implemented.

**Methods/Approach:**

The PEPPA framework (**P**articipatory, **E**vidence-based, **P**atient focused **P**rocess for **A**dvanced practice nursing) illustrates the steps in the process of developing and implementing the FNP role in a country. These steps include: determining the need for the role, deciding on a model of care, developing/implementing the curriculum, relevant policies, and scope of practice (SOP), and integrating the role into relevant nursing regulations and Ministry of Health (MOH) guidelines and documents.

**Outcomes:**

The assessment has been completed, a locally tailored competency-based FNP curriculum has been developed, revised, and implemented, the FNP SOP has been approved and MOH guidelines are being updated to reflect current evidence-based practice and to integrate the FNP role. Continuous cycles of improvement/revision were needed to adapt the curriculum and SOP to meet local needs. Clinical placements were challenging since this is a new health cadre, but most challenges were overcome and many resulted in important opportunities for interdisciplinary collaboration.

**Summary:**

Outcomes from this quality improvement initiative demonstrate that it is feasible to develop and implement a locally responsive, competency-based FNP program in a low resource setting and enroll students, despite time and financial constraints. Adapting the curriculum and SOP from western countries can provide a foundation for program development but revision to assure that the program is responsive to local context is then needed. There is general acceptance of the role among Eswatini communities and professional stakeholders with emphasis on the need for FNP graduates to be clinically competent and able to function independently. Policy work related to deploying new graduates is ongoing.

## Introduction: Why Did We Introduce the FNP Role in Eswatini?

### Description of the local problem

The first graduate level advanced practice nursing program in the Kingdom of Eswatini (formerly Swaziland) was launched in 2017 as a strategic response to the health needs of the population. Eswatini occupies approximately 1.700 km^2^ in the southern easterly parts of sub-Sahara Africa, and has a population of about 1.3 M, more than 80% of whom live in rural settings. Maternal and child mortality is high. Whilst the country’s efforts to manage and control the HIV burden has had good results, rates of HIV and MDR-TB remain high [1]. In addition, there is an escalating burden of non-communicable diseases (NCD) accounting for 37% of all deaths [2,3]. This double burden of high morbidity and mortality and an emerging epidemic of NCD, in the context of a dwindling health workforce, both in numbers and skill, skewed towards urban areas despite the majority of the population dwelling in rural areas, requires new ways of thinking and new ways of delivering care [4].

### Background

In 2014 the Ministry of Health released the national strategic plan detailing how it intends to ensure all Emaswati have access to promotive, preventive, curative, and rehabilitative health services of sufficient quality [5]. Health sector activities to attain this goal included a scaling up of interventions and a focus on introducing interventions as and where needed. There are five levels of care in Eswatini organized into three service delivery systems: referral (national referral hospital, regional referral hospital); primary care (health centre, public health unit, clinics A and B); and individual/household/community (community) [5]. The strategic plan envisions changing the health system from a siloed/disarticulated, disease-focused system to a client-centred system that views individuals holistically across the biopsychosocial domains [5]. The 2014–2018 strategic plan is in the process of review and revision but in the interim the existing document continues to guide the country’s health policy [5].

The advanced practice nurse, with a patient/family-centred, holistic approach to care, is uniquely well suited to help build capacity for this expanded vision of health service delivery in Eswatini. The role has been shown to be effective in improving access to, and quality of, services, which has implications for Eswatini in achieving its strategic health goals. The nurse practitioner role was innovated in the United States as an advanced practice nursing role for registered nurses who obtained additional education and clinical experience to prepare them to assess, diagnose, treat, and educate patients in a holistic manner. Nurse practitioners focus on health promotion and disease prevention and provide care for people who experience common acute and chronic problems, along with complex and long-term care needs; skills which align well with the call to action for care across the lifespan outlined in the Eswatini national strategic plan. An abundance of evidence supports positive, cost effective outcomes, and patient satisfaction with care provided by nurse practitioners [6,7,8,9,10]. At least 20 countries have adopted the advanced practice nurse practitioner (NP) role [11,12,13,14,15,16,17,18,19]. A relevant study from South Africa examined the effectiveness of an NP-MD collaborative care model for the treatment of MDR-TB. It demonstrated that clinics which adopted this care model had better treatment outcomes compared to other clinics, and that patients treated by the NP or MD had similar outcomes [20]. The Eswatini strategic plan provides a firm foundation on which to introduce the APN role and build capacity for universal health care in the country [5,21].

Of note, an earlier educational program introduced the family nurse practice role in Swaziland (1979–1995). This was a one-year certificate program through a partnership between the University of Swaziland and Denmark. The program was provided to diploma-educated nurses and was not recognized as an advanced practice nurse role. While helpful in its time, the role lacked official recognition (grade and pay differential) and expanded scope of practice. These factors, along with the lack of continuing education, were significant barriers to optimal role function and contributed to the decision to develop the family nurse practice role as a master’s level advanced practice nurse practitioner role [22].

### Purpose of the innovation/improvement

Nurses are the backbone of Eswatini’s human resource for health. In response to the health care demands of the population, constrained available resources and strategic priorities of the MOH nursing leaders collegially unified their efforts to establish and implement an advanced practice graduate level nursing program as one of the ways to achieve and sustain universal health care. Key stakeholders included, academia (University of Eswatini; UNESWA), the Eswatini Ministry of Health (MOH) and the Eswatini Nursing Council (ENC). UNESWA was established in 1982 with a mandate to assist with the national development effort through manpower production in priority areas including nursing. The FNP program, the first master’s program within the faculty of health sciences, resides within the General Nursing Department under the direction of the Head of Department. The MOH endeavours to improve the health of the people of Eswatini by providing leadership in the production and delivery of health services that increase the longevity and quality of life. The Chief Nursing Officer plays an important role in advising the ministry and government in terms of the contribution of nursing to achieving the MOH mission, as well as advising them in terms of training needs in the nursing cadre. She is critical to assuring that the advanced practice nurse’s role is integrated into the healthcare cadres recognized by the ministry, as well as the various treatment guidelines promulgated by the MOH. The regulatory body (ENC) regulates and sets the standards for nursing and midwifery education and practice, as well as professional conduct for the benefit of stakeholders. The Registrar provides executive leadership for the implementation of strategies, objectives, and decisions of the ENC Board within the framework of the delegated authorities, values, and policies of the Council being guided by the Nurses and Midwives act of 1965.

Taken together, these three institutions are the drivers of the advanced practice role in Eswatini. Fortunately, a mechanism to facilitate inter-sectoral (academia, ministry of health, nursing council, practice) communication among nursing leaders in the country existed and they meet on a regular basis to discuss issues which affect nursing.

## Approach: What Did We Do?

### Context

**Figure 1:**
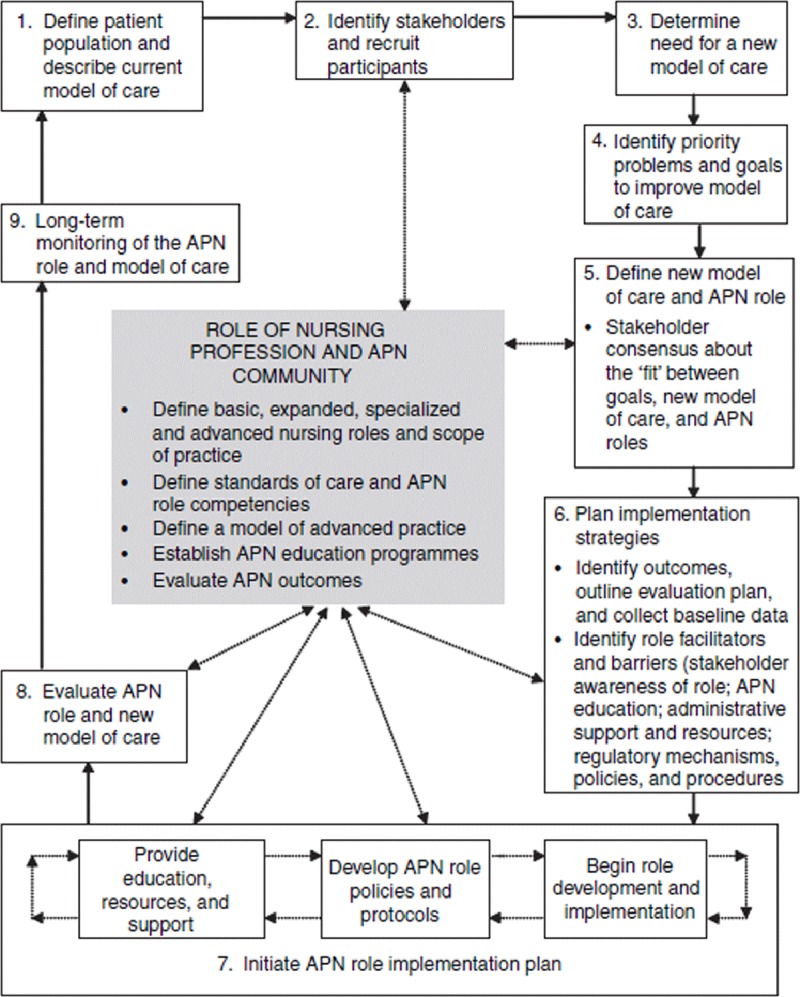
The PEPPA Framework [Bryant-Lukosius, D., & DiCenso, A. (2004). A framework for the introduction and evaluation of advanced practice nursing roles]. Reprinted with permission.

The PEPPA framework (Participatory, Evidence-based, Patient focused Process for Advanced practice nursing) provides a context to consider the essential, interconnected roles of education, policy, regulation, and practice in the development and implementation of the family nurse practitioner role in Eswatini [23]. (See Figure 1.) The National Health Sector Strategic Plan (2014) established the need and rationale for establishing this new cadre of health worker including; defining the patient population, describing the current model of care, identifying stakeholders, determining the need for new models of care delivery, and identifying priority problems and goals to improve models of care (Figure 1: Steps 1 through 4) [5]. In advance of this Ministry of Health document, concerns about critical unmet health needs of everyday Emaswati led UNESWA to systematically assess the country’s health care needs. Stakeholder meetings with policy, practice, and community experts were conducted (2004–2007) and the findings mirrored the major findings in the National Health Sector Strategic Plan, but also provided important insights about the shortage of human resources for health, particularly specialized skills in nursing services [24].

In view of the high burden and increasing complexity of disease, constrained resources (material and human), and the challenges of serving the rural populace, UNESWA established a graduate level advanced practice Family Nurse Practitioner (FNP) program that focused on strengthening primary care and increasing access to universal health care [24]. In collaboration with relevant policy makers (MOH, ENC) and informed by local health policy and the international literature, UNESWA embraced developing and implementing this new cadre for nursing to accelerate progress toward universal health coverage. (Refer to Figure 1: Step 5.)

In keeping with the logical sequence of activities outlined in the PEPPA framework, the next steps were related to defining the nursing role, educational preparation, and policy/regulatory requirements. (Refer to the middle box in Figure 1.) The Eswatini Nursing Council (ENC) had an existing set of nursing standards, scope of practice, and competencies which defined the basic, expanded, specialized, and master’s nursing roles; however, a scope of practice (SOP) for the master’s level advanced practice nursing role was not included. This is an important early step since the SOP provides the authority to work independently at the advance practice level; an important distinction from the task sharing model which has become common in Africa. A scope of practice for the FNP role taken from the U.S. was initially adopted by the nursing council and subsequently revised to be more locally relevant as part of a national initiative, supported by the WHO, to update the SOP for all levels of nursing in Eswatini. A technical working group that included representatives from the training institutions, supporting partners, and practice reviewed relevant references and FNP curriculum presented a draft scope to stakeholders and integrated stakeholder feedback into the final SOP, which was approved by the ENC board.

The Ministry of Health is currently integrating the FNP role into several important policy documents, including the Standard Treatment Guidelines and Essential Medications [25]. The university’s administration approved the Family Nurse Practitioner Programme and curriculum in 2016 after the ENC had reviewed and approved of the same. The first students were enrolled in August of 2017.

### The Master’s Family Nurse Practitioner Curriculum

The program proposal submitted to the university included the curriculum. Faculty completed curriculum templates which included a course description, course objectives, expected learning outcomes, course content, teaching and learning activities, and assessment measures. Faculty, the nursing council, the University Senate, and other key stakeholders reviewed the templates to assure the courses were designed for the graduate level and would provide FNPs with the didactic and clinical knowledge, skills, and abilities required for successful FNP practice. The Eswatini FNP scope of practice, in tandem with the National Organization of Nurse Practitioner Faculties competency areas (Scientific Foundation, Leadership, Quality, Practice Inquiry, Technology and Information Literacy, Policy, Health Delivery Systems, Ethics, Independent Practice), informed the competencies for the UNESWA FNP curriculum to assure that the program would graduate practice-ready FNPs at the advanced practice level [26].

### Refining the curriculum, course content and facilitating delivery

Initially the program was conceptualized as a two-year full-time program, but multiple logistical challenges emerged that necessitated moving to a three-year part time curriculum designed for the working nurse. Coursework was clustered in the first two years with the bulk of clinical time occurring in an internship over two semesters in the last year of the program. Classes were originally scheduled one day a week (alternating Friday/Saturday) over the twelve-week semester, but students found this difficult due to work/time conflicts and attendance was poor. Students and faculty strategized on the best format and agreed on two one-week blocks (0800–1700 Monday through Friday) and five Saturdays. Faculty will sometimes hold additional Saturdays if time is needed to review material in depth or take tests. The one-week blocks allowed students to apply for annual leave and attendance improved, although everyone agreed that the long days were exhausting and limited their ability to absorb information between lectures. Another limitation to the block system is that if a student misses a block due to illness or work issues, they miss one-third of the content which is difficult to make up. Additionally, an unintended consequence of moving the program to part-time is that it eliminated the opportunity for students to be included in their facility work plan, which impacted their ability to have approved time off and financial support from the MOH for FNP education. However, on balance, the block/Saturday system has allowed nurses to enroll in and advance through the program despite the real-life scheduling and financial constraints students face. The program of study and course sequence for the FNP Programme is outlined in Table 1.

**Table 1:** Program of Study, Master’s Family Nurse Practitioner Programme, University of Eswatini.

Semester I	Semester II

GNS 603 Pathophysiology	GNS 605 Pharmacotherapeutics Across the Life Span
GNS 609 Health Economics	GNS 611 Monitoring and Evaluation
GNS 690 Seminar on Issues for the Family Nurse Practitioner	GNS 614 Human Resource Management
	GNS628 Advanced Health Assessment [24 clinical hours]
**Semester III**	**Semester IV**

GNS 601 Research Methods in Nursing	GNS 612 Data Analysis and Interpretation
GNS 607 Family Nurse Practice for the Child and Adolescent [36 clinical hours]	GNS 618 Family Nurse Practice for Adults and Elders [36 clinical hours]
GNS 620 Clinical Teaching in Nursing	GNS 622 Common Mental Health Disorders in Children and Adults
**Elective Courses**^†^	**Elective Courses**^†^
GNS 635 Demography for Health Sciences	GNS 632 Health Informatics
GNS 637 Theories in Nursing	GNS 634 Analysis of Health Policy Issues
GNS639 Palliative care	GNS 636 Emerging Issues in Health
	GNS 638 Biostatistics for EBP
**Semester V**	**Semester VI**

GNS 613 Internship [264 clinical hours*]	GNS 613 Internship [254 clinical hours*]
GNS 699 Master’s Thesis	GNS 699 Master’s Thesis

* Internship hours: 528 total over Semester V and VI are divided into 216 hours for adulst and elders; 192 hours for children and adolescent; 120 hours for mental health. Total clinical hours for the program: 624. Total credits: 58. ^†^ Students choose one elective in semester III and IV for a total of two elective courses.

Advanced health assessment was not part of the FNP program at the outset because the differences in pedagogy and competencies between undergraduate and graduate level health assessment were not fully understood by the university. Once it because clear that the UNESWA FNP program would not be on par with FNP training requirements in countries with longstanding experience with the advanced practice role unless they had the three core APN courses (advanced pathophysiology, pharmacology, and health assessment), the faculty decided to add a course that focused on advanced physical/health assessment, critical thinking, and differential diagnosis. An advanced health assessment course was developed and approved by the university as an addition to the curriculum. The course has been implemented and both faculty and students agree that the knowledge and skills emphasised in the course provides an important foundation for their subsequent clinical courses.

As the courses began to be rolled out, continuous pedagogic enhancements were necessary to assure that students would be clinically competent at the advanced practice level. Lectures were kept to a minimum with much of the class time transitioned to case-based discussion related to the weekly course content and health problems the students encountered in their clinical placements and workplace. Students practiced concisely and accurately presenting a clinical problem using a standardized communication approach in both the written and oral format. As RNs, most students were accustomed to delivering episodic, disease-focused care, and treating acute/urgent problems. Even though students’ pre-licensure education introduced them to holistic care, what they experienced in practice was different. It was difficult for students to conceptualize care that was holistic, family-centred, and focused on health promotion or disease prevention. Situating learning in clinical scenarios they understood was important. For example, having the students examine the Eswatini HIV guidelines and identify care strategies across the biopsychosocial domains; and focusing on health promotion at every visit allowed students to conceptualize and assimilate the philosophy of holistic care across the patient care experience.

The course texts were initially drawn from U.S. advanced practice nursing textbooks, which provide important insights into the pathophysiology of disease and the foundation for evidence-based advanced practice nursing [27,28,29]. Although these texts provided the technical expertise needed as well as the FNP-focus, there was a need to add locally relevant context and treatment guidance for the common health problems seen in Eswatini [30,31,32,33,34,35,36,37,38]. Most local/regional texts adopted emanated from South Africa and the Eswatini MOH guidelines provided another rich resource for the students. (Table 2) Identifying locally relevant texts and guidelines took a great deal of in-country and transnational networking and attending local/regional primary care conferences. The health policy course became a natural place to introduce the importance of universal health care and assuring that every Liswati has access to available, affordable, effective health care. The Chief Nursing Officer was invited to co-lecture in this course as a way to engage students in the policy process and identify ways that future FNPs can be involved in shaping policy that integrates the FNP in building capacity for universal health coverage.

**Table 2:** Examples of Ministry of Health Documents that are integrated into the course content.

Standard Treatment Guidelines	Standard Treatment Guidelines and Essential Medications List of Common Medical Conditions in the Kingdom of Swaziland (2012, currently being revised)

	Children’s Protection and Welfare Act (2012)
HIV	Swaziland Integrated HIV Management Guidelines (2018)
	Amendment to the 2018 Eswatini Integrated HIV management guidelines (2019)
	Major changes to the 2018 Integrated HIV Guidelines (2019)
Hypertension	Hypertension Guidelines
Immunizations	National Immunization Schedule (2018)
Malnutrition	National Guidelines on the Integrated Management of Acute Malnutrition (IMAM). (2015)
Palliative Care	Kingdom of Swaziland Ministry of Health National Palliative Care Clinical Guidelines (2018)
	Introduction to Children’s Palliative Care: A Multidisciplinary Course for Professionals Trainee Manual (2016)
	Introduction to Children’s Palliative Care: A Multidisciplinary Course for Professionals Facilitators Manual (2016)
Psych	Intervention Guidelines for Managing Common Psychiatric Conditions (2015)
STD	Swaziland National Sexually Transmitted Infections Guidelines (2018)
TB	National Tuberculosis Control Programme of the Kingdom of Eswatini (2019)

A challenge that is perhaps unique to clinical master’s programs in Eswatini is the competing priority to complete a master’s research thesis whilst trying to work and complete clinical hours. It has been a struggle for students to meet the university master’s thesis requirement and gather primary data in addition to their clinical practice hours. Most programs in the U.S. acknowledge that the generation of knowledge through research may not be feasible in a clinical master’s program and propose instead a model for translating evidence to practice (improvement science). In this model students engage in appraising evidence and applying it to practice in the form of a quality improvement project. Improvement science situates the students’ scholarship in practice, can be done concomitant with clinical hours and contributes to accelerating translation of evidence to practice.

### Establishing effective clinical placements: Challenges and opportunities

Given that this is a new role in the country, it only stands to reason that there would be challenges in establishing effective clinical experiences for the students; however, this also created some exciting opportunities. The challenges fall into three categories; lack of understanding of the role and scope of practice of the FNP, lack of clarity around administrative procedures, and student scheduling issues.

Because the advanced practice role at the master’s level is new to the country, there are no master’s prepared NPs to serve as preceptors. This meant the faculty had to turn to medical doctors to serve as preceptors. The CNO was instrumental in working with the MOH to request physician preceptors for the FNP students. Many doctors had little knowledge about the role, although some had been exposed to the role in other countries and many had master’s degrees and were familiar with the educational process. Most doctors were receptive to being preceptors, were eager to learn what was expected of them, and welcomed the opportunity to help train FNPs who they saw as future colleagues in high stress settings that are severely understaffed and under-resourced. Preceptors are given a packet including information about the FNP role, FNP curriculum and competencies, an array of clinical learning tools and a preceptor checklist. Some physicians requested clarification from the MOH that they could spend their time precepting nurses. Guided by the CNO, the MOH sent a letter to all health centers and hospitals clarifying the FNP precepting role of the physician.

Administratively, there were challenges in how to approach potential placements and how to optimize the learning environment. Initially it was not clear whether faculty needed to negotiate the placement with the Matron or the Medical Director, but it became apparent that both needed to be consulted. Although we were seeking physician preceptors, the nursing administration needed to be informed of the role of nursing graduate students in their facilities since the usual custom is that Eswatini nurses are under nursing administrations’ jurisdiction. As graduate students, all FNP students are registered nurses and are identified as such in the clinical site. Generally, nursing administration was accepting of the students’ presence, although some were concerned that nursing would be responsible for their actions. The administration was assured that these students would work under the supervision of physicians since they were seeking advanced diagnostic and treatment skills.

Student scheduling constraints presented challenges for clinical as it did for coursework. All of the students work full time, many in remote areas. Most wanted the clinical practicum to be in their worksite, which presented its own unique set of challenges and opportunities. Students were provided as much flexibility as possible allowing students to create their own schedule, do a portion of their hours at their worksite, as long as it was not on working time, and completing clinical hours on the weekends, as long as they had a preceptor on duty. There were several areas of concern to be considered when students used their worksite for clinical hours. They may be pressed into working when staffing is short, and they may hesitate to ask questions (be a student) in a setting where they are usually considered the expert. Guidelines for completing student clinical hours at one’s worksite were developed and disseminated. Some experiences were scheduled for sites outside their workplaces to facilitate students’ mastering the full range of competencies required by the curriculum. If, for example, students practiced in a rural setting, they might be expected to spend some hours at a national referral hospital and/or specialty site (e.g. HIV clinic). Similarly, students who work in a national referral hospital will be expected to spend some time in rural areas.

In addition to the challenges, developing and implementing the clinical placements also created several exciting opportunities. Family Medicine doctors have been quick to welcome the FNP students to the field of Family Practice and to involve the students in developing this specialty. They have also been outstanding, engaged, preceptors with high expectations of the students. Doctors practicing in remote area have been eager to teach the students a wide array of interventions that they provide in areas isolated by geography and/or resources. The clinical placements have also provided an important opportunity for faculty and students to engage in interprofessional collaboration, an important skill for newly minted FNPs and their colleagues and an important measure of the ability of different health care cadres to work together toward a common purpose.

Efforts are ongoing to find the right balance between practical considerations and effective learning environments and to assure that all graduates of the FNP program have the advanced practice competencies, clinical expertise, and role development to effectively engage as an FNP.

### Faculty readiness and development

Assuring a mix of academically and clinically qualified faculty to teach in the FNP program is essential to the success of preparing practice-ready FNPs who can deliver high quality, evidence-based care. UNESWA was fortunate to have a full complement of PhD prepared faculty able to teach the didactic content, but needed to scale up its clinical advanced practice expertise; which makes sense given that the role is new to Eswatini. When developing the program, one faculty member was supported to travel to Botswana for two years to pursue a master’s degree as an FNP. She returned to UNESWA as a qualified FNP, but being new to the role was not quite ready to step into a leadership position in the program. The university needed experienced FNP faculty who could teach the clinical courses, direct the clinical experiences, and mentor UNESWA’s newly minted FNP faculty member to the role. To meet this need, the Global Health Service Partnership (GHSP: a partnership between the U.S. Peace Corps, PEPFAR, and Seed Global Health) seconded experienced U.S. nurse practitioner faculty to serve for a year as visiting faculty with the intention of supporting the FNP program until a core of clinically experienced UNESWA FNP faculty could be developed and become self-sufficient. At the completion of her service, one GHSP faculty stayed as a UNESWA faculty member and assumed the role of program coordinator providing much needed continuity and expertise and other essential contributions to fully develop the program. Additionally, through a Fulbright Specialist Award, a visiting faculty specialist has supported the program through teaching and leadership by participating in the block, consulting on curriculum, serving as a research advisor, and supporting the program faculty as needed. The goal is for UNESWA to be self-supporting in both the didactic and clinical areas within the next three years.

### Assessing readiness to introduce the master’s FNP role in the country

Once the program began to enroll students and the ENC approved the scope of practice, attention turned to assessing readiness to integrate the advanced practice FNP role into the Eswatini health system (Figure 1: Step 6). This provided an excellent opportunity to re-introduce the role to the general population of Eswatini nurses, physicians, community members, leaders, and policy makers and solicit their feedback on strategies for successful integration. Stakeholder meetings were carried out in the four regions of the country and yielded feedback and suggestions relevant to education, practice, and policy.

Stakeholder meetings continued to be generally supportive of the FNP role; specifically the value of the role in increasing access to comprehensive services at the primary care level, thus reducing the need for referrals (or speeding the referral process when needed) and helping to push the queue. They also saw the value of the FNPs being from the community and able to speak the language. Most physicians are foreigners and do not speak siSwati, necessitating the nurses to take time from their duties to translate. They saw the cultural and linguistic concordance of native Emaswati FNPs being of value both to patient care and their role as nurses.

In relation to practice and policy, stakeholder feedback focused on the need for the FNP to be able to function autonomously at the advanced practice level. They raised practical issues, such as assuring there was a consult room and equipment for the FNP, as well as administrative and policy considerations, including having a defined job description, established reporting/personnel mechanism (will they report to nursing or medicine), the need for the FNP to have a defined grade/pay, and the need for revised standard treatment guidelines, which integrate the scope of FNP practice. There was some concern about how the FNP would differ from the RN who already has many responsibilities for serving as the first point of contact for patient care. Some RNs were reluctant to enroll in the advanced practice FNP program unless there was a new cadre and remuneration commensurate with the increased responsibility.

In advance of deploying newly minted FNP graduates, stakeholders stressed the importance of raising awareness of the scope of practice for this role, including outreach to the community, patients, nurses, physicians, and health care facilities. They saw the need for deployed FNPs to have access to continuing professional development and support, especially during their first year of practice.

### Outcomes: What we learned

The initiative to date has demonstrated that a locally relevant, master’s level, advanced practice education program can be implemented in this resource-constrained setting and be aligned with the national health priorities and integrated into nursing’s scope of practice, the national health strategic plan and the national human resources for health scheme. Currently there are three cohorts enrolled and the first cohort graduates in October 2020. Through the development and early implementation phase, it is evident the curriculum format needs to be flexible, allowing for part-time study in a country with a dire shortage of nurses who cannot be released from their duties to attend post-graduate school full time. The process of refining coursework is iterative and involves continuous cycles of improvement and course content changes that respond to challenges and successes. Proficiency as a clinician needs to be emphasized and be context appropriate integrating regional and in-country resources in addition to western advanced practice textbooks. Contextualizing course content, particularly teaching cases, is necessary to fit the Eswatini health care environment.

Students need high level, mentored, locally relevant clinical experiences and FNP faculty who can guide them through both didactic and practical experiences. Establishing meaningful clinical placements is challenging but manageable and provides opportunities to build cross-disciplinary relationships particularly with family medicine physicians. These cross-disciplinary relationships are critical to the role being accepted by nursing and interprofessional colleagues. The MOH is critical to creating and supporting preceptor partnerships.

Intersectoral collaboration, mapped out in the PEPPA Framework, was essential from the beginning to assure successful development and implementation of the role. To actualize the vision of building capacity for universal health care the FNP Programme needed to produce practice-ready clinicians who will be autonomous advanced practice providers prepared to assess, diagnose, and treat health conditions at the point of care. The ENC needed to approve a scope of practice that allows the FNPs to practice at an APN level. Non-African models for FNP scope of practice can assist in getting a program started, but need to be adapted to fit the local African context. As new services are decentralised and introduced at different levels, the Standard Treatment Guidelines and Essential Medicines List of Common Medical Conditions will facilitate availability of the relevant commodities and medicines at the appropriate levels [25]. Revision of the 2012 standard treatment guidelines is currently underway at the Ministry of Health and includes FNP faculty representation [25].

### Discussion: What it means

The need to build capacity to improve access to universal health care in Eswatini is clear and compelling; achieving this vision requires new ways of thinking and delivering care. The advanced practice nursing role has been shown to improve access to care that is equitable, affordable, acceptable, and effective and adding the advanced practice FNP to the cadre of health providers in Eswatini holds promise to strengthen health care across the lifespan and accelerate progress towards universal health care. Intersectoral collaboration among policy, education, and regulation proved essential throughout the steps of the process including; identifying gaps in the current health system, determining the need for a new cadre, agreeing on a model that will achieve the desired outcome, and working together to operationalize the vision. The Eswatini experience demonstrates that developing the role is indeed feasible in a resource constrained setting and that the different health sectors can work together.

As the first cohort prepares to graduate, important work remains to be done. The university needs to develop an evaluation of the educational program and practice outcomes, and prospectively follow students during their first few years of practice. The MOH needs to establish a job description that includes the grade and pay for family nurse practitioners, develop a human resources for health deployment scheme, prepare health workers and the public to accept these new providers, and continue to update treatment guidelines to include the FNP role. The ENC needs to prepare to register the first Eswatini trained FNPs to practice.

Education, policy, regulation, and practice are inextricably connected. No one part of this initiative to development and implement the FNP role in Eswatini can stand alone and when components are implemented singly, the results are suboptimal and may have unintended consequences. Excellent educational programs, high level policy and regulatory standards developed in silos may result in FNPs who cannot practice to the top of their license and do not have a job when they graduate. Conversely if regulatory bodies approve the authority to practice in the advanced practice role without graduates to fill these roles or if the ministry promulgates standard treatment guidelines with no providers to implement them at the point of care, the common goal of increasing access to care will not be achieved. Emaswati who currently lack access to basic health care deserve to have the relevant stakeholders collaborate towards a common goal; better health for all Emaswati. The first FNP program graduates will be the ones to pave the way and demonstrate the value of the role in improving health.
